# PARADIGM-2: Two parallel phase I studies of olaparib and radiotherapy or olaparib and radiotherapy plus temozolomide in patients with newly diagnosed glioblastoma, with treatment stratified by MGMT status

**DOI:** 10.1016/j.ctro.2017.11.003

**Published:** 2017-11-21

**Authors:** Ben Fulton, Susan C. Short, Allan James, Stefan Nowicki, Catherine McBain, Sarah Jefferies, Caroline Kelly, Jon Stobo, Anna Morris, Aoife Williamson, Anthony J. Chalmers

**Affiliations:** a Institute of Cancer Sciences, University of Glasgow, UK; b Leeds Institute of Cancer and Pathology, University of Leeds, UK; c Beatson West of Scotland Cancer Centre, NHS Greater Glasgow & Clyde, Glasgow, UK; d Christie NHS Foundation Trust, Manchester, UK; e Addenbrooke’s Hospital, Cambridge University Hospitals NHS Foundation Trust, Cambridge, UK

**Keywords:** Glioblastoma, Poly(ADP-ribose) polymerase, Olaparib, Radiotherapy, O6-methylguanine methyltransferase, Dose escalation, Radiosensitizer

## Abstract

Glioblastoma has a dismal prognosis and molecular targeted agents have failed to improve outcomes to date. PARADIGM-2 is a phase I dose escalation study evaluating olaparib plus radiotherapy ± temozolomide in newly diagnosed glioblastoma, using MGMT methylation status to stratify patients and inform treatment schedules.

## Highlights

• The manuscript details the rationale, design and protocol for PARADIGM-2.
• PARADIGM-2 comprises two parallel phase I, dose escalation studies of the PARP inhibitor olaparib in combination with radiotherapy (for MGMT unmethylated patients) and radiotherapy-temozolomide (for MGMT methylated patients) in newly diagnosed glioblastoma.
• This is a novel approach to phase I dose escalation trial design that maximises the potential for patients with glioblastoma to benefit from the addition of the radio- and chemosensitizing drug olaparib.

## Background

Glioblastoma (GBM) is the most common primary malignant brain tumour and carries the worst prognosis: median survival is 12–18 months and 2-year survival rates approximately 25% [2]. Incidence is increasing, with over 2-500 cases diagnosed in the UK each year [1]. Patients aged under 70 with World Health Organisation (WHO) performance status 0–1 are treated with debulking surgery where possible followed by radical radiotherapy with concomitant and adjuvant temozolomide. Benefit from temozolomide is limited to patients whose tumours exhibit methylation of the MGMT promoter region [3], and the acceptability of withholding temozolomide from patients with MGMT unmethylated tumours in the context of clinical trials has been validated. This approach is particularly valuable when an investigational agent is expected to increase the toxic effects of temozolomide, or vice versa.

Attempts to improve outcomes in GBM by increasing radiation dose have been unsuccessful, so recent trials have intensified chemotherapy or added molecular targeted agents to standard radio-chemotherapy. The negative results of multiple phase II and III trials support the viewpoint that molecular targeted agents inhibiting specific signaling pathways are generally ineffective against this heterogeneous disease [4], [5], [6]. Furthermore, the measurable incidence of late neurotoxicity in these patients indicates that strategies aimed at intensifying treatment must be robustly tumour specific [7], [8], [9].

PARADIGM-2 aims to improve treatment of GBM patients by combining conventional radio-chemotherapy with pharmacological inhibition of the DNA damage response (DDR) using olaparib, on orally bioavailable inhibitor of poly(ADP-ribose) polymerase (PARP). PARP is an abundant nuclear protein involved in detection and repair of DNA breaks induced by ionising radiation or DNA alkylating agents such as temozolomide. It is consistently over-expressed in GBM [10] but barely detectable in the normal brain. The radiosensitising effects of PARP inhibition have been demonstrated in multiple glioma cell lines [11], and are observed only in actively dividing cells. Since normal brain tissue is composed almost exclusively of non-dividing cells, combining radiotherapy with PARP inhibitors is predicted to enhance its therapeutic ratio [12], [13], [14], [15]. In contrast, combining PARP inhibition with temozolomide is known to exacerbate dose-limiting haematological toxicity [16], [17]. However our recent dose escalation study of olaparib plus temozolomide in patients with recurrent GBM showed that intermittent olaparib (three days per week) can safely be combined with full dose temozolomide given daily for 42 days. We will therefore introduce olaparib on an intermittent schedule in the MGMT methylated patient group. For patients in the MGMT unmethylated group, omission of temozolomide will enable continuous olaparib treatment and facilitate dose escalation with minimal risk of haematological toxicity.

PARP activation is also strongly implicated in the pathogenesis of neuroinflammation: numerous studies have shown that inhibition of PARP protects the healthy brain from a range of neurotoxic insults [18]. Since radiation-induced neuroinflammation is a key contributor to the acute, subacute and late phases of neurotoxicity [19], we hypothesized that olaparib might protect the normal brain from the adverse effects of radiation and it was agreed that a four week period of olaparib treatment post-radiotherapy was justified.

## Methods and study design

PARADIGM-2 study is co-sponsored by the University of Glasgow and NHS Greater Glasgow and Clyde. It is funded by Cancer Research UK (CRUK), The Brain Tumour Charity and AstraZeneca under the terms of the Combinations Alliance collaboration between the CRUK Centre for Drug Development and the Experimental Cancer Medicines Network (ECMC). The study has received National Research Ethics Committee approval.

**Fig. 1:**
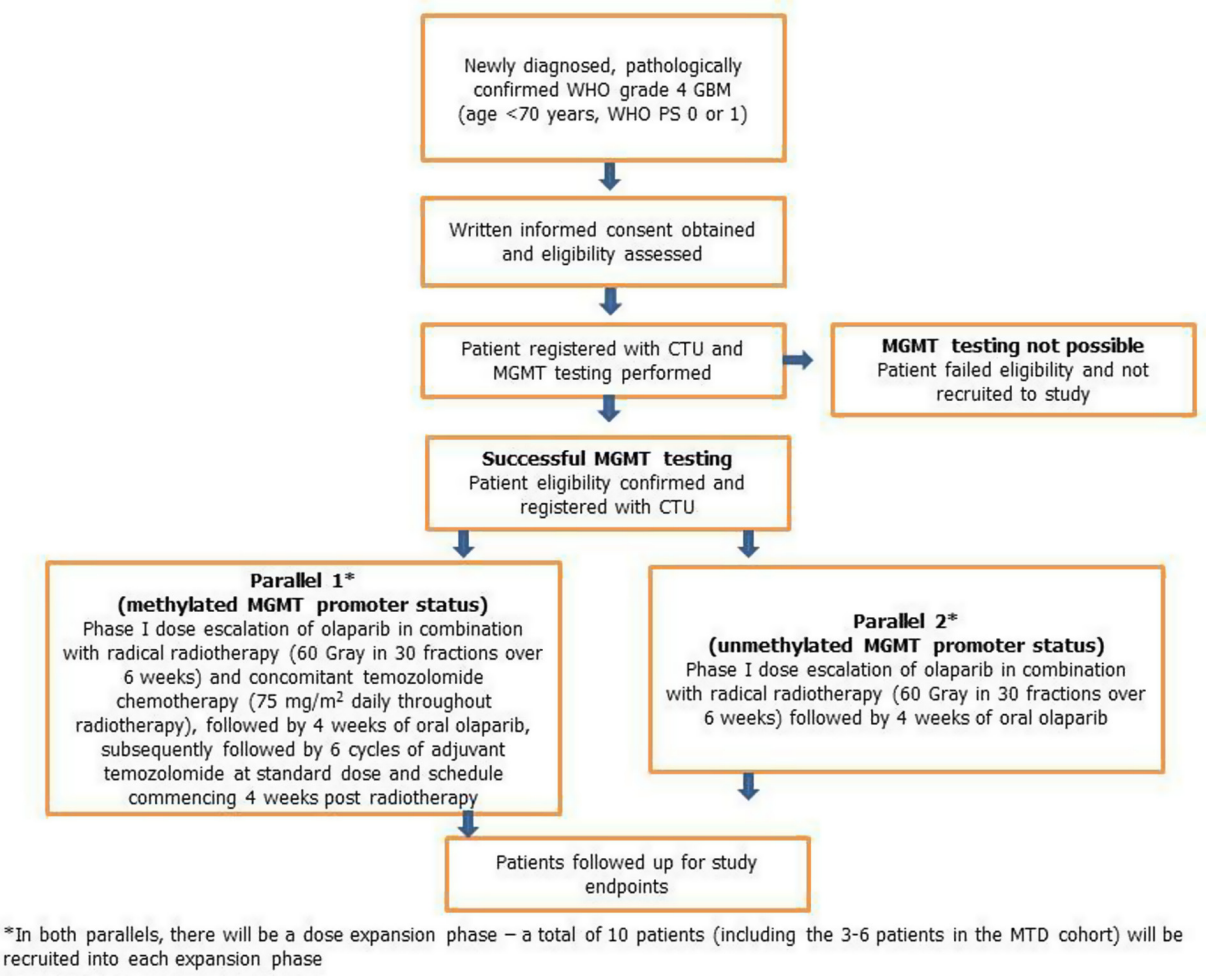
Study design for PARADIGM-2.

This is a multi-centre, open-label, non-randomised, dose-escalation phase I clinical trial of olaparib in combination with radiotherapy, with or without temozolomide, in patients with newly diagnosed GBM. There are two parallel groups within one trial protocol (Fig. 1).

Patients allocated to parallel 1 (MGMT methylated) will receive standard radical radiotherapy (60 Gy in 30 fractions over 6 weeks) and concomitant temozolomide chemotherapy (75 mg/m^2^ daily throughout radiotherapy) in combination with olaparib, followed immediately by a further 4 week period of olaparib given at the same dose and schedule as during radiotherapy. Olaparib treatment will be intermittent and the daily dose and schedule will be escalated based on a 3 + 3 phase I design. Patients will then receive adjuvant temozolomide as per standard care. The dose escalation strategy is shown in Table 1.

**Table 1:** Dose escalation strategy for MGMT methylated patients.

Dose level	Olaparib Dose starting day 1 of radio-chemotherapy and continuing until 4 weeks after end of radio-chemotherapy	Temozolomide Dose starting day 1 and continuing daily for 6 weeks of radio-chemotherapy
−1	50 mg single dose Day 1 of each week	75 mg/m^2^ once daily
1	100 mg single dose Day 1 of each week	75 mg/m^2^ once daily
2	100 mg once daily Days 1 and 2 of each week	75 mg/m^2^ once daily
3	100 mg once daily Days 1–3 of each week	75 mg/m^2^ once daily
4	150 mg once daily Days 1–3 of each week	75 mg/m^2^ once daily
5	150 mg once daily Days 1–4 of each week	75 mg/m^2^ once daily
6	150 mg once daily Days 1–5 of each week	75 mg/m^2^ once daily

Patients allocated to parallel II of the study (MGMT unmethylated) will receive radical radiotherapy (60 Gy in 30 fractions over 6 weeks) in combination with olaparib, followed immediately by a further 4 weeks of olaparib at the same dose. Olaparib treatment will be continuous and the daily dose will be escalated based on a 3 + 3 phase I design. Patients in parallel II will receive neither concomitant nor adjuvant temozolomide; the dose escalation strategy for olaparib is shown in Table 2.

**Table 2:** Dose escalation strategy for MGMT unmethylated patients.

Dose level	Olaparib dose starting three days prior to radiotherapy and continuing until 4 weeks afterwards
1	50 mg once daily, continuous
2	100 mg once daily, continuous
3	100 mg twice daily, continuous
4	200 mg twice daily, continuous

PARADIGM-2 targets a population of patients with newly diagnosed GBM who meet the NICE TA23 eligibility criteria for treatment with radical radiotherapy and concomitant temozolomide. Approximately 44–68 patients across both parallels will be recruited. Potential patients will be identified at neuro-oncology multi-disciplinary team meetings in participating centres.

### Rationale for study design

Several randomised phase III trials have demonstrated the prognostic and predictive value of MGMT promoter region methylation as a robust molecular biomarker in GBM [2], [5], [6]. Patients whose tumours exhibit methylation of the MGMT promoter region have better prognosis and derive clinical benefit from the addition of temozolomide to radiotherapy. In contrast, patients whose tumours exhibit unmethylated MGMT promoter regions have poorer prognosis and derive no benefit from the addition of temozolomide to standard radiotherapy [3], [20]. The MGMT unmethylated group represents approximately 55% of cases and is a population with particularly pressing unmet clinical need. Current clinical practice is to treat all patients with concomitant and adjuvant temozolomide, regardless of MGMT status. In the context of a clinical trial, however, it is widely accepted that temozolomide can be safely and ethically omitted in patients with MGMT unmethylated tumours, as long as an alternative treatment is offered [21]. Several commercial and investigator led studies have demonstrated the feasibility and acceptability of this approach.

In PARADIGM-2, therefore, treatment will be informed by MGMT methylation status. Patients whose tumours have unmethylated MGMT promoter regions will be treated with radiotherapy and olaparib, without temozolomide. This approach liberates these patients from the haematological toxicity associated with olaparib-temozolomide combination therapy, allowing continuous daily olaparib dosing in tandem with daily radiotherapy fractions. Patients with MGMT methylated tumours derive clear benefit from temozolomide and will receive olaparib with radiotherapy in addition to concomitant and adjuvant temozolomide.

*Inclusion criteria* for PARADIGM-2 study

• Age < 70 years
• Histologically confirmed diagnosis of glioblastoma (WHO grade IV)
• WHO performance status 0 or 1
• Sufficient tumour material for MGMT promoter methylation assay
• Life expectancy greater than 12 weeks
• No previous radiotherapy for primary or secondary CNS malignancy
• Adequate haematological, hepatic and renal function
• Able to commence radiotherapy treatment within 6 weeks (±1 week) of surgery
• Willingness to comply with scheduled visits, treatment plans, laboratory tests and other trial related procedures
• Able to swallow trial medications
• Evidence of non-child bearing status for women of child-bearing potential

*Exclusion criteria* for PARADIGM-2 study

• Active concomitant malignancy
• Prior treatment for primary or secondary CNS malignancy
• Confusion or altered mental state that would prohibit understanding and giving of informed consent
• Any previous treatment with a PARP inhibitor, including olaparib
• Any red blood cell transfusions within 28 days
• Patients with myelodysplastic syndrome or acute myeloid leukaemia
• Patients with uncontrolled seizures

### Study objectives and end-points

The overall hypothesis is that combining olaparib with radiotherapy ± temozolomide will improve outcomes for patients with newly diagnosed GBM, without significantly exacerbating toxicity.

The primary objective of parallel I (MGMT methylated) is to establish the safety, toxicity, maximum tolerated dose (MTD) and optimum schedule of olaparib in combination with radical radiotherapy and concomitant temozolomide in patients with newly diagnosed GBM, with which to proceed to a randomized phase II study.

The primary objective of parallel II (MGMT unmethylated) is to establish the safety, toxicity, MTD and optimum schedule of olaparib in combination with radical radiotherapy in patients with newly diagnosed GBM, with which to progress to a randomized phase II study.

Secondary objectives for both parallels are:

• Define dose-limiting toxicities associated with olaparib in combination with radiotherapy ± temozolomide.
• Obtain preliminary information on the impact of this regime on acute and sub-acute neurotoxicity
• Obtain preliminary evidence of efficacy of the regimens, in terms of progression-free survival and overall survival.

Toxicity will be recorded using the Common Terminology Criteria for Adverse Events (CTCAE) scoring system, version 4.03. Definitions of dose limiting toxicity are listed in Table 3.

**Table 3:** Definitions of dose limiting toxicities.

*MGMT methylated and unmethylated patients*
Failure to complete radiotherapy because of toxicity, as considered by the investigator in consultation with the safety review committee (SRC) (i.e. with no evidence of tumour progression).
Any grade ≥ 3 non-haematological toxicity (see appendix CTCAE 4.03) that was not present prior to commencing olaparib and which, in the opinion of the investigator in consultation with the SRC, is due to olaparib or the combination of olaparib and radiotherapy (±temozolomide). Such toxicities will be classed as a DLT from start of olaparib treatment until the end of olaparib treatment (ie. 4 weeks after the end of radiotherapy treatment).
Neutropenia grade 4that persists for ≥5 days and occurs during the olaparib treatment period.
Febrile neutropenia grade ≥ 3 (absolute neutrophil count <1.0 × 109/L and fever ≥38.5 °C) that occurs during the olaparib treatment period.
Thrombocytopenia grade 4 which persists for ≥ 5 days or is associated with active bleeding or requiring platelet transfusion and occurs during the olaparib treatment period.
Failure to complete >75% of planned olaparib treatment at the prescribed dose because of toxicity.

*MGMT methylated patients only*
Failure to complete concomitant temozolomide chemotherapy because of toxicity, as considered by the investigator in consultation with the SRC (i.e. with no evidence of tumour progression).
Any toxicities grade ≥3 occurring during adjuvant temozolomide will not be considered a DLT. However any toxicity grade ≥3 deemed due to interaction between temozolomide and olaparib will be considered a DLT. The evaluable DLT period ends after cycle 1 of adjuvant temozolomide.

Patients recruited into the trial will be invited to authorize use of blood samples and surplus tumour tissue for translational research projects. The main aims of these projects will be to identify molecular biomarkers with potential to predict which patients will benefit from the addition of olaparib to radiotherapy ± temozolomide.

### Radiotherapy Quality Assurance

Radiotherapy Quality Assurance (RTQA) in PARADIGM-2 will be conducted through the UK RTTQA team. Pre-trial participation questionnaires will be sent to participating sites to collate information on staffing, equipment and delivery technique details. Each site will then be required to submit a ‘dummy-run’ case, comprising a case chosen by the site that has been outlined and planned as per trial protocol. Radiotherapy dosimetry and treatment data will be collected on all patients participating in the trial.

### Duration of trial participation

*Parallel I:* patients will be assessed 4 weeks after the final fraction of radiotherapy, at which point they will commence adjuvant temozolomide, during which they will be assessed every 4 weeks. Upon completion or discontinuation of temozolomide, patients will enter follow-up and be assessed every 3 months or until clinical or radiological disease progression. Once date of progression has been recorded, patients will be followed up locally to establish overall survival.

*Parallel II:* patients in parallel II will be assessed 4 weeks after the final fraction of radiotherapy and then a further 8 weeks later, after which they will be assessed every 3 months or until clinical or radiological progression. After progression, patients will be followed up locally to establish overall survival.

### Trial analysis plan

Dose escalation of olaparib in both parallels will follow a 3 + 3 cohort design but also take into account data from the current and all previous cohorts, enabling dose escalation decisions to be informed by late emerging adverse events. The study will explore cohorts until the MTD or recommended dose and schedule of olaparib has been determined.

*Parallel I:* Up to 6 cohorts of 3–6 patients. The final cohort will be expanded to include 10 patients. The estimated sample size is 25–40 patients.

*Parallel II:* Up to 4 cohorts of 3–6 patients. The final cohort will be expanded to include 10 patients. The estimated sample size is 19–28 patients.

Data analysis of data will be descriptive, summarizing toxicities and dose-limiting toxicities by dose escalation cohort. Details of treatment delivery will be summarized and worst grade of toxicity during olaparib treatment tabulated. Secondary endpoints (overall survival and progression-free survival) will be summarized by individual cohort using techniques appropriate to the number of patients available. Data from expansion cohorts will be presented as Kaplan-Meier analyses.

## Discussion

Previous attempts at radiotherapy dose escalation have failed to demonstrate clinical benefit [4], [5], [6] for patients with GBM and there remains a clinical need to improve outcomes. PARADIGM-2 offers a novel approach to previous studies in this area by combining a potent, tumour specific radiosensitising agent with conventional radiotherapy or radiochemotherapy. Another novel aspect is the use of MGMT promoter region methylation status to individualise treatment. The DNA damage response targeting approach adopted in this study also avoids the potential pitfalls of targeting individual oncogenic signaling pathways, which has been shown to be ineffective in this heterogeneous and genetically complex disease [7].

Multiple phase III trials have demonstrated the prognostic and predictive value of MGMT promoter region methylation status in patients with GBM [2]. The design of PARADIGM-2 aligns with recent clinical trials that have demonstrated the acceptability of omitting temozolomide in patients with MGMT unmethylated tumours [21]. This reduces the risk of haematological toxicity and will enable continuous olaparib dosing with a view to maximising tumour control.

Patients will be asked to consent to collection of surplus tissue for translational research. These translational studies will exploit the research team’s expertise in DNA damage response (DDR) biology to undertake preliminary evaluation of a panel of candidate predictive biomarkers. Based on published data demonstrating that the radiosensitising effects of olaparib are enhanced in the presence of DDR defects, this research will evaluate correlations between clinical outcomes and a panel of Immunohistochemical markers of DNA replication and cellular proliferation. Further translational end points will focus on the correlation of genomic instability with DDR defects and the novel, unpublished observation that primary cell lines derived from genomically unstable tumours show enhanced radio-sensitisation responses to PARP inhibitors.

If the olaparib-radiotherapy combination is tolerated by MGMT unmethylated patients and the MTD of olaparib is determined to be 100 mg twice daily or greater, we will aim to proceed to a randomized phase II study in this patient population. If the olaparib-radiotherapy-temozolomide combination is tolerated in MGMT methylated patients and olaparib can safely be delivered on at least three days per week we will aim to proceed to a randomized phase II study in this population.
